# Conservation and Sustainable Development of Rice Landraces for Enhancing Resilience to Climate Change, with a Case Study of ‘Pantiange Heigu’ in China

**DOI:** 10.3390/life16010143

**Published:** 2026-01-15

**Authors:** Shuyan Kou, Zhulamu Ci, Weihua Liu, Zhigang Wu, Huipin Peng, Pingrong Yuan, Cheng Jiang, Huahui Li, Elsayed Mansour, Ping Huang

**Affiliations:** 1 Institute of Food Crops, Yunnan Academy of Agricultural Sciences, Kunming 650201, China; sy_kou@yaas.org.cn (S.K.); lwhynu@yaas.org.cn (W.L.); wuzhigangswu@163.com (Z.W.); lihuahuiweida@163.com (H.L.); 2 Bureau of Agriculture and Rural Affairs, Weixi Lisu Autonomous County 675000, China; 11227066lm@sina.com (Z.C.); php0806@sina.com (H.P.); 3 Department of Crop Science, Faculty of Agriculture, Zagazig University, Zagazig 44519, Egypt

**Keywords:** agrobiodiversity, sustainable agriculture, climate resilience, crop breeding, food security, traditional varieties, phenotypic traits, molecular genetics, nutritional quality, Pantiange Heigu

## Abstract

Climate change poses a threat to global rice production by increasing the frequency and intensity of extreme weather events. The widespread cultivation of genetically uniform modern varieties has narrowed the genetic base of rice, increasing its vulnerability to these increased pressures. Rice landraces are traditional rice varieties that have been cultivated by farming communities for centuries and are considered crucial resources of genetic diversity. These landraces are adapted to a wide range of agro-ecological environments and exhibit valuable traits that provide tolerance to various biotic stresses, including drought, salinity, nutrient-deficient soils, and the increasing severity of climate-related temperature extremes. In addition, many landraces possess diverse alleles associated with resistance to biotic stresses, including pests and diseases. In addition, rice landraces exhibit great grain quality characters including high levels of essential amino acids, antioxidants, flavonoids, vitamins, and micronutrients. Hence, their preservation is vital for maintaining agricultural biodiversity and enhancing nutritional security, especially in vulnerable and resource-limited regions. However, rice landraces are increasingly threatened by genetic erosion due to widespread adoption of modern high-yielding varieties, habitat loss, and changing farming practices. This review discusses the roles of rice landraces in developing resilient and climate-smart rice cultivars. Moreover, the Pantiange Heigu landrace, cultivated at one of the highest altitudes globally in Yunnan Province, China, has been used as a case study for integrated conservation by demonstrating the successful combination of in situ and ex situ strategies, community engagement, policy support, and value-added development to sustainably preserve genetic diversity under challenging environmental and socio-economic challenges. Finally, this study explores the importance of employing advanced genomic technologies with supportive policies and economic encouragements to enhance conservation and sustainable development of rice landraces as a strategic imperative for global food security. By preserving and enhancing the utilization of rice landraces, the agricultural community can strengthen the genetic base of rice, improve crop resilience, and contribute substantially to global food security and sustainable agricultural development in the face of environmental and socio-economic challenges.

## 1. Introduction

Rice (*Oryza sativa* L.) is one of the most important field crops worldwide [1]. It provides a primary source of calories and nutrition for more than half of the global population [2]. Rice cultivation supports the livelihoods of millions of smallholder farmers in many societies, particularly across Asia and Africa [3]. The global cultivated area of rice is approximately 168 million hectares, which produces about 800 million tons [4]. Its demand continues to increase due to population growth and changing diets [5]. However, rice productivity faces significant challenges caused by biotic and abiotic stresses, which are increased by current climate change [6].

Climate change poses a substantial threat to rice production through an increasing frequency and intensity of extreme weather events [7]. The primary climatic drivers are rising temperatures and altered precipitation patterns. These fluctuations directly translate into specific physiological and ecological stresses for rice cultivation [8]. Extreme temperatures lead to spikelet sterility, reduced grain filling, and yield loss. Moreover, warmer conditions can accelerate pest lifecycles and expand the geographical range of both insects and pathogens. The shifts in precipitation regimes are characterized by more intense rainfall events and prolonged dry periods, which increase the risks of both flooding and drought stress. These biotic and abiotic pressures challenge the stability of rice production systems. However, most of the modern rice varieties lack the resilience to biotic and abiotic stresses [9]. Conversely, rice landraces represent rich genetic potential adapted to diverse agro-ecological conditions [10]. They are traditional local varieties that have been cultivated by farmers for centuries [11].

The preservation of local rice landraces is a critical strategy for global food security and climate resilience. These genetically diverse populations have been enhanced by centuries of natural and farmer selection within specific agro-ecosystems. These varieties constitute an irreplaceable repository of adaptive traits [12]. The landraces possess inherent tolerance to abiotic pressures such as drought, salinity, temperature extremes, and poor soil fertility, as well as resistance to evolving pests and diseases. The current climate change intensifies these stresses and threatens the genetic uniformity of modern high-yielding cultivars [13]. Landraces offer essential genetic raw material for breeding the next generation of climate-smart rice. Furthermore, their nutritional superiority is often rich in essential micronutrients, antioxidants, and proteins, addressing growing concerns over dietary quality and hidden hunger. Therefore, conserving these varieties is fundamental to broadening the genetic base of rice. This effort protects biodiversity and cultural heritage and also ensures the adaptive capacity and nutritional integrity of our food systems for future generations facing unprecedented environmental uncertainty [14].

**Figure 1:**
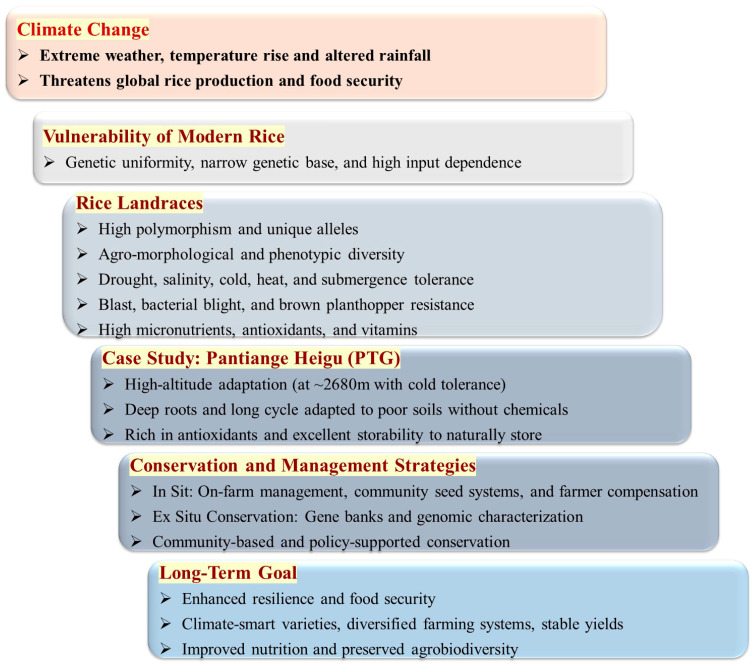
Framework for the integrated utilization and conservation of rice landraces under climate change.

Rice landraces are threatened by genetic erosion due to the cultivation of modern varieties, changes in agricultural practices, and urbanization [15]. The loss of landraces causes a narrowing of the genetic base essential for future improvement [16]. The conservation and sustainable development of rice landraces are crucial for enhancing the adaptive capacity of rice production systems to face the challenges of climate change worldwide. This review discusses the importance of rice landraces, genetic diversity, and their unique characteristics under current climate change (Figure 1). Using the Pantiange Heigu (PTG) landrace, cultivated at one of the highest altitudes globally in Yunnan Province, China, as a case study, it explores effective integrated conservation and sustainable development practices. Therefore, this review advances the current literature by providing a novel integrated synthesis of rice landraces with a case study. It illustrates a practical, multi-stakeholder framework for conservation. This systematically combines in situ and ex situ strategies with policy support, market-based valorization, and community engagement. This framework is clarified through an in-depth case study of PTG landrace. By demonstrating how genetic resource conservation can be directly linked to climate resilience strategies and socio-economic development, this review offers a transferable model for conservation agrobiodiversity in face of global environmental change.

## 2. Genetic Diversity of Rice Landraces

**Figure 2:**
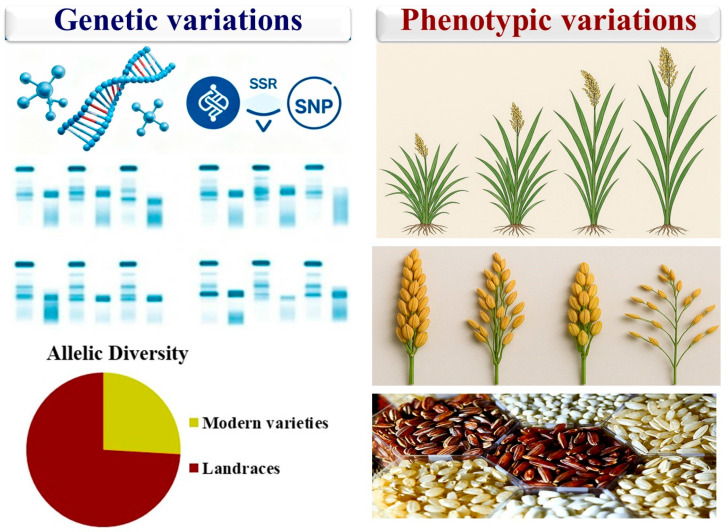
Landraces exhibit rich genetic diversity and phenotypic diversity in agro-morphological traits and grain characteristics.

Rice landraces exhibit wide genetic variation reflecting their long-term adaptation to diverse agro-ecological zones [17]. This genetic diversity includes wide variations of molecular, morphological, and biochemical characteristics. The foundation of landrace resilience lies in their genetic diversity from molecular variation to expressed phenotypic traits (Figure 2). The genetic diversity at the molecular level includes polymorphisms in Simple Sequence Repeats (SSRs) and Single Nucleotide Polymorphisms (SNPs), as well as the presence of unique alleles. This genetic variation is directly expressed as diverse agro-morphological characteristics and grain quality. Therefore, the broad genetic base conserved within landrace populations directly translates into the wide range of observable traits. These traits confer tolerance to biotic and abiotic stresses and enhance nutritional value. At the molecular level, rice landraces exhibit high polymorphism across multiple loci with distinct alleles [18]. Molecular marker analyses demonstrate high polymorphism and genetic heterogeneity in different rice landrace populations [19]. This diversity is crucial for breeding programs to improve rice resilience for future climatic challenges. Many studies reported that rice landraces have greater genetic diversity compared to modern cultivars. Hour et al. [17] assessed the genetic diversity in 148 rice accessions comprising wild rice, landraces, and modern cultivars from different countries. The results identified a total of 953 alleles presenting high genetic diversity. The genetic analyses ranked wild rice with the highest diversity, followed by landraces, while the modern cultivars showed the least diversity. The detected polymorphism and unique alleles in landraces were absent in the modern cultivars. Genetic erosion was observed in modern japonica cultivars more than in indica ones, caused by narrow selection focused on grain quality. Although japonica modern cultivars had reduced variation, the japonica landraces showed higher genetic variation than the indica landraces. Ahmadi et al. [20] assessed rice landrace populations that were collected over 30 years in West Africa. This study evaluated two collections from diverse agro-ecological zones from tropical forests to Sudanian savannah. The first group contained 442 accessions while the second group included 776 accessions. All accessions were genotyped with SSR and SNP markers to assess genetic diversity patterns. A subset of 600 accessions was genotyped by sequencing using ApeKI enzyme and Illumina HiSeq 2000 platform. Phenotyping for days to heading was conducted on separate upland and lowland field trials in Guinea for 239 Collect-1 and 412 Collect-2 accessions. The meteorological data displayed significant climate changes in the study areas, including increased temperatures and altered rainfall patterns. Detected flowering genes and quantitative trait loci (QTLs) such as *OsGI*, *Hd1*, and *OsphyB* in the genotyped accessions indicated the genetic basis of adaptation. The adaptive potential detected is attributed to the genetic structure of rice landraces and the rich genetic diversity of the rice meta-population. This in situ evolution illustrates the value of maintaining diverse landrace populations for resilience to climate variability. Deepika et al. [21] evaluated genetic diversity in diverse rice landraces using SSR markers. The polymorphism information content values varied widely across markers. Dendrogram analysis grouped the landraces into ten diverse clusters, indicating high genetic diversity.

At the phenotypic level, landraces also possess diverse morphological traits in plant height, leaf area, tillering capacity, panicle branching, panicle length, number of grains, grain weight, grain shape, grain color, and aroma [22]. The variations in plant height can affect lodging resistance and light interception. Leaf area influences photosynthetic capacity, and tillering ability determines the number of productive tillers. The panicle structure varies significantly in branching patterns and panicle length. Grain characteristics such as number, weight, shape, color, and aroma differ widely among rice landraces. Certain aromatic landraces possess high nutritional and medicinal value, including high levels of antioxidants, vitamins, and microelements. High levels of bioactive compounds improve dietary diversity, which plays an important role in enhancing food security. These phenotypic traits contribute to agronomic performance, cultural identity, and market differentiation. Gautam et al. [23] studied genetic diversity in traditional rice landraces from the Andaman Islands. This study classified the evaluated landraces into four diverse groups based on 22 agro-morphological and biochemical characters. Significant variations were detected in important characters such as content of flavonoid and anthocyanin, and grain yield. These variations, due to natural selection, are valuable to improve nutrition, yield, and resilience to environmental stresses. Suman et al. [24] explored the phenotypic diversity in rice landraces. The assessed landraces displayed significant variations in plant height, days to flowering, grain yield components, grain size, and nutritional quality. In addition, they applied molecular analyses to study genotypic diversity within rice landraces. The results detected high polymorphism across multiple loci and the presence of unique alleles and gene combinations. Islam et al. [25] investigated 48 rice landraces to study genetic variability and trait associations. The results displayed significant genetic variability in 14 agro-morphological traits among assessed rice landraces, including plant height, flag leaf area, grain number per panicle, grain length, kernel length, and thousand-grain weight. The results reflected a broad genetic base adapted to local agro-ecological conditions. Correlation and path coefficient analyses demonstrated that days to first flowering, filled grain number per panicle, grain weight, grain length, and kernel length positively influence grain yield. These results demonstrated the importance of these agro-morphological traits in assessed rice landraces to maintain agricultural biodiversity and support climate resilience.

## 3. Distinctive Characteristics of Rice Landraces

**Figure 3:**
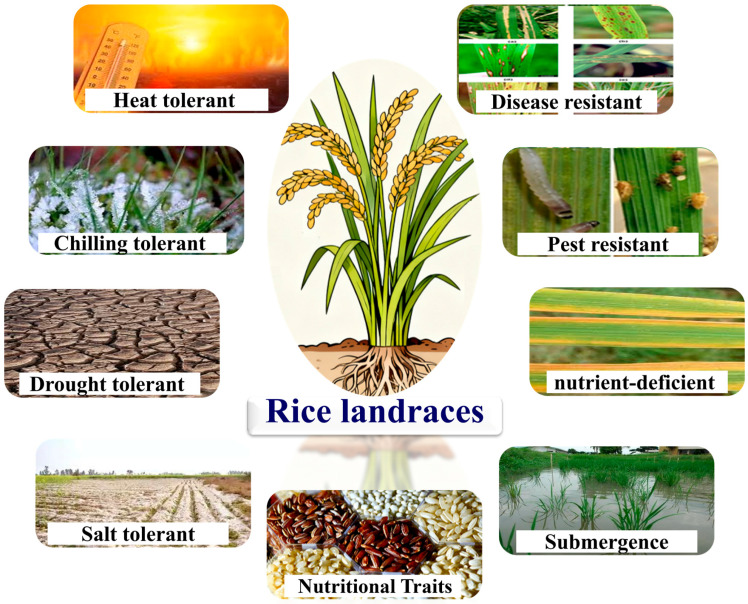
Rice landraces provide favorable alleles to improve defense against biotic and abiotic stresses with enhanced nutritional quality.

Rice landraces possess complex gene combinations associated with tolerance to different abiotic stresses such as drought, salinity, temperature extremes (heat and cold), submergence, and nutrient deficiency (Figure 3). Moreover, many landraces possess diverse alleles related to resistance to biotic stresses, including pests and diseases. In addition, rice landraces exhibit great grain quality characters including high levels of essential amino acids, antioxidants, flavonoids, vitamins, and micronutrients. Rice landraces also display diverse grain characteristics and storability. Muhu-Din Ahmed et al. [26] evaluated 102 rice genotypes including landraces under high-temperature field conditions. The objective of this study was to identify heat-tolerant genotypes and determine characteristics associated with heat tolerance in rice. The obtained results identified specific landraces as highly heat-tolerant genotypes. The identified heat-tolerant landraces were based on favorable characteristics that correlated strongly with stable yield under heat stress. The identified heat-tolerant landraces exhibited favorable plant height, leaf area, panicle length, and chlorophyll content, grain weight, spikelet fertility, and grain-filling capacity under heat stress. This study demonstrated that rice landraces possess favorable alleles that can be exploited in developing climate-resilient rice varieties for warming climate. Song et al. [27] evaluated 150 rice landraces under low temperature conditions (10 °C). Also, they applied high-density SNP markers (67,511 SNPs) to detect candidate genes for cold tolerance. Phenotypic evaluation indicated a wide range of variations in survival under cold stress. These genetic variations exhibited significant differentiation between indica and japonica subpopulations. Japonica genotypes exhibited greater cold tolerance than indica genotypes. GWAS was conducted to detect quantitative trait loci (QTLs) linked to cold tolerance. The results of GWAS located multiple significant loci associated with cold tolerance in the assessed landraces. Expression analysis of the candidate genes under cold treatment validated their functional roles in modulating cold tolerance. These detected genes in the assessed rice landraces are promising for improving cold tolerance in rice breeding programs under current climate change.

Beena et al. [28] demonstrated the significant genetic potential in rice landraces for drought tolerance. This study evaluated 300 rice landraces from different origins. Approximately 700,000 single-nucleotide polymorphisms (SNPs) were applied across the rice genome. Phenotypic evaluation was conducted under well-watered and drought stress conditions in field trials during two growing seasons. Leaf rolling, canopy temperature, plant height, days to flowering, grain yield, and drought sensitivity index were recorded. The results displayed significant genetic variation among the assessed rice landraces for drought tolerance under drought stress conditions. Twenty-three rice landraces exhibited superior drought tolerance based on multiple indices. A total of 340 significant SNP markers associated with important traits such as grain yield, plant height, days to flowering, leaf rolling, and drought sensitivity indices. The detected SNP markers across rice chromosomes demonstrate the complex polygenic nature of drought tolerance. Moreover, candidate genes close to significant SNPs are associated with root architecture and osmotic adjustment. Consequently, this study demonstrates the importance of rice landraces as an essential source of adaptive alleles to develop drought-resilient rice varieties.

Shanmugam et al. [29] evaluated 119 genotypes comparing 115 landraces and four modern varieties under submergence conditions. Early-stage submergence was applied by submerging seedlings under 10 cm of water for 15 days. Anaerobic germination percentage, anaerobic vigor index, shoot and root length, number of leaves and roots, grain length, grain breadth, length-breadth ratio, and seed weight were recorded. In addition, the response index was estimated to compare growth under stress to control conditions. In addition, molecular analysis was applied using gene-specific markers linked to anaerobic germination tolerance. The assessed genotypes exhibited significant differences in anaerobic germination percentage and anaerobic vigor index. Several landraces displayed high tolerance levels exceeding the tolerant checks. Moreover, shoot and root length, number of leaves and roots, and seed physical characteristics were superior in the identified tolerant landraces. This demonstrates the adaptive responses of landraces compared to modern varieties under submergence conditions. Furthermore, molecular marker analysis detected allelic variation at key loci associated with anaerobic germination tolerance among the assessed genotypes. The molecular results confirmed the presence of favorable alleles of submergence tolerance in the landraces. Consequently, this study identified promising landraces that possess potential alleles for improving tolerance to submergence in flood-prone environments.

Lokeshkumar et al. [30] assessed 145 rice genotypes under salinity conditions. The evaluated genotypes comprised 100 landraces and 45 advanced breeding lines. These genotypes were evaluated in controlled hydroponic system under saline stress conditions (EC 10.0 dS/m) and non-saline control conditions (EC1.2 dS/m). Genetic diversity and haplotype analysis focused on the *Saltol* QTL region associated with salinity tolerance. Simple Sequence Repeat (SSR) markers linked to the *Saltol* locus were used to genotype the assessed genotypes. Stress injury scores, shoot and root growth, and ion accumulation were recorded. Phenotypic evaluation under saline conditions displayed significant differences in tolerance levels among the evaluated genotypes. The rice landraces exhibited superior performance under salinity conditions. The identified salt-tolerant landraces displayed lower injury scores, better shoot and root growth, and effective ion balance under stress conditions. Their performance indicated an important source of adaptation that can be exploited in improving resilience of rice in salt-affected regions. The molecular haplotype analysis of the *Saltol* QTL region demonstrated that the identified landraces possess unique alleles associated with enhanced salt tolerance. These unique alleles were absent in advanced breeding lines. The combination of superior morphophysiological performance and favorable haplotypes demonstrates the potential of these landraces for breeding salt-tolerant rice varieties adapted to saline environments.

Rao et al. [31] evaluated 472 rice genotypes comprising landraces and breeding lines under recommended and low nitrogen conditions. Also, this study employed a modern genomics approach to identify the potential genes associated with tolerance to low-nitrogen. Grain yield, nitrogen uptake, and nitrogen utilization efficiencies were recorded under low-N stress. The results identified more than 100 rice landraces with superior root architecture, chlorophyll content, nitrogen translocation, and grain yield under low-N stress. The identified landraces could be considered candidates for enhancing nitrogen uptake, nitrogen translocation, and grain yield under low nitrogen stress. Moreover, association mapping located 12 genomic regions associated with yield-related traits under low-N stress. Four significant regions identified stable QTLs for low-N yield. These QTLs are associated with nitrogen uptake, assimilation, and remobilization. This study identified promising landraces for enhancing nutrient efficiency under low nitrogen conditions. The detected genomic regions provide targets for marker-assisted breeding to enhance low-N tolerance in high-yielding cultivars. This strategy is essential for developing varieties with lower fertilizer requirements to support sustainable rice production.

Raj et al. [32] evaluated 182 rice landraces for blast resistance. The tested landraces were inoculated under controlled greenhouse with virulent isolates of *Magnaporthe oryzae*. Lesion type, size, and distribution were recorded to identify resistant, moderately resistant, and susceptible landraces. Moreover, molecular marker analysis was applied to detect the presence of blast resistance genes. The results revealed substantial variation in blast resistance among the assessed landraces. Phenotypic screening under greenhouse conditions identified several landraces with strong and moderate resistance to *Magnaporthe oryzae*. Also, molecular marker analysis detected the presence of multiple resistance genes in these landraces. Some landraces displayed combinations of major *R* genes that contribute to durable resistance. Population structure analysis for these landraces showed wide distribution of resistance genes. The findings of this study demonstrated the value of these landraces for rice breeding to develop durable and broad-spectrum blast-resistant rice varieties.

Zhao et al. [33] evaluated 200 rice landraces for bacterial blight resistance. In addition, this study applied specific PCR using functional markers of four major resistance genes. The objective was to classify the assessed landraces into resistant, susceptible, or heterozygous based on allele presence. The results identified several rice landraces with strong resistance to bacterial blight due to the presence of the *Xa21* and *Xa7* genes. These landraces demonstrated durable resistance against diverse strains of the bacterial blight pathogen. Resistant landraces showed limited disease symptoms and enhanced growth under pathogen challenge. The presence of *Xa21* and *Xa7* genes provided durable resistance and enhanced the plant defense system before and during infection by *Xanthomonas oryzae*. The genotypes that lack *Xa21* and *Xa7* genes were susceptible to bacterial blight. Furthermore, the genotypes carrying ineffective genes *xa5* and *xa13* were also susceptible to bacterial blight. This study identified promising rice landraces carrying effective resistant genes to enhance genetic diversity for resistance to bacterial blight.

Roy et al. [34] evaluated 218 rice landraces for resistance to brown planthopper *Nilaparvata lugens* under greenhouse and field conditions. Number of insects per plant and percentage of chaffy grains were studied. The results displayed significant variation in resistance to brown planthopper in the evaluated rice landraces. Forty landraces exhibited low damage scores and higher percentages of healthy grains. These forty landraces were examined for antixenosis, antibiosis, and biochemical parameters related to host-plant resistance. These landraces showed effective antixenosis and antibiosis. Moreover, biochemical analysis demonstrated higher levels of phenolic compounds in these resistant landraces. Correlation analysis displayed strong associations between biochemical parameters and resistance to brown planthopper. Hence, this study identified rice landraces with durable resistance to brown planthopper for breeding programs aimed at developing resistant varieties.

Rice landraces equally serve as an indispensable resource of genetic variation for enhancing nutritional quality. This nutritional superiority comes from the accumulation of diverse alleles governing the biosynthesis and sequestration of essential micronutrients, proteins, and health-promoting phytochemicals. These traits are often diminished during intensive breeding for yield and uniformity in modern varieties. The genetic mechanisms reinforcing this enhanced nutritional profile are multifaceted. These genotypes possess elevated expressions of metal transporter genes, which lead to superior accumulation of iron and zinc in the grain endosperm [35]. Furthermore, they contain natural variants in genes involved in the anthocyanin and flavonoid biosynthesis pathways. The pigmented red or black grains possess high antioxidant capacities. Moreover, bioactive compounds like flavonoids and phenolic acids contribute to plant defense mechanisms against abiotic stresses and pests. Furthermore, many landraces also exhibit favorable profiles of essential amino acids, including lysine, which is typically limited in cereals. In this context, Parikh et al. [36] evaluated traditional rice landraces for grain nutritional and quality traits. Grain length, width, and weight, micronutrient contents, protein content, and antioxidant capacity were recorded. The results revealed significant variability among assessed landraces for grain nutritional and quality traits. Considerable variation was detected in micronutrient content, especially iron and zinc. In addition, grain physical attributes and biochemical parameters showed wide variability. Principal component and cluster analyses classified the assessed landraces into distinct clusters based on their nutritional and quality profiles. Some landraces exhibited high concentrations of iron, zinc, protein content, and antioxidants. These identified landraces are promising donors for improving micronutrient deficiencies through rice breeding. In addition, Jukanti et al. [37] evaluated nutritional and quality traits in 345 rice landraces, using advanced statistical methods including mixed clustering and selection indices to identify superior genotypes. This work comprehensively profiled grains for physical characteristics (length, width, and shape), nutritional components (protein content, amylose, and micronutrients), and functional qualities (antioxidant capacity, and flavonoid and phenolic content). The findings indicated substantial variations among landraces. Several landraces exhibited excellent combinations of high micronutrient density, elevated antioxidant activity, and favorable cooking properties. The mixed clustering approach successfully identified distinct groups of landraces optimized for specific quality functions, such as high-nutrition pigmented rice or premium aromatic varieties. Furthermore, the selection index methodology pinpointed specific landraces that serve as ideal donors for biofortification breeding, possessing balanced trait profiles. These landraces could be exploited to improve nutrition without compromising agronomic performance. Therefore, this work emphasizes that traditional landraces represent a vital source of diverse quality traits.

## 4. Case Study: “Pantiange Heigu” Landrace in Yunnan, China

### 4.1. Characteristics of Pantiange Heigu Landrace

**Figure 4:**
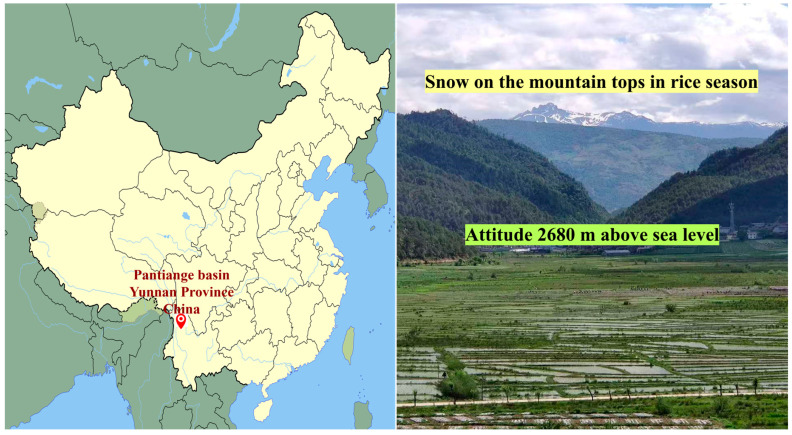
Geography of the high-altitude Pantiange basin in Yunnan Province, China, with cold fields at elevations up to 2680 m.

Pantiange Heigu landrace (PTG) is cultivated in Pantiange basin, located in Weixi County, Diqing Prefecture, Yunnan Province, China (Figure 4). This region covers an area of about 282 square kilometers and is characterized by high mountains and deep valleys. Cultivation fields in Pantiange are located at elevations up to 2680 m above sea level. This location is one of the highest-altitude rice-growing areas worldwide. The climate is characterized by cold temperatures with an average annual temperature of around 10 °C. Soils in this region are loose, highly porous, moisture-rich, and deficient in nutrients. The PTG landrace is adapted to this region, despite these severe conditions.

**Figure 5:**
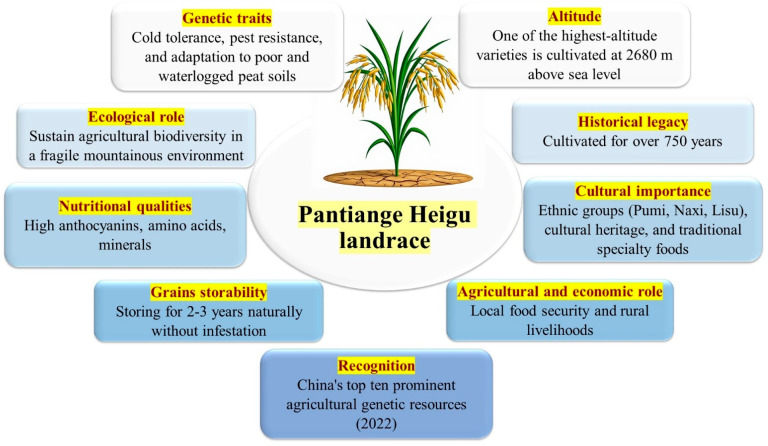
Genetic, nutritional, ecological, and cultural importance of Pantiange Heigu (PTG) landrace in Pantiange, Yunnan, China.

The PTG is one of the highest-altitude traditional rice varieties cultivated globally. It has been grown for over 750 years by Pumi, Naxi, and Lisu communities. PTG is japonica rice with a mean plant height of 100.6 cm and extended leaves with small angle. Its root system extends to depths up to 12 cm. The mean panicle length ranges from 17.4 cm with a mean of 79.6 grains per panicle and mean 1000-grain weight of 30.61 g [38]. It has a prolonged growth cycle of approximately 181 days [39]. This landrace exhibits tolerance to cold temperatures, resistance to pests, and adaptation to nutrient-poor and waterlogged peat soils. It is traditionally cultivated without pesticides or chemical fertilizers, depending on its resilience to environmental stresses [40]. Its yield is relatively lower compared to modern high-yielding varieties, while it provides stable grain production under adverse conditions for other cultivars. It has been certified as a Grade-A Green Food product [41]. Hence, PTG is considered an important genetic resource for breeding stress-tolerant varieties (Figure 5).

PTG provides high levels of anthocyanins, amino acids, bioactive compounds, and essential minerals [42]. Its aromatic grains are rich in amino acids such as glutamic acid and lysine, as well as polyphenols and flavonoids [43]. The flavonoid content is significantly higher in the modern rice varieties. It is characterized by black hulls and red kernels. It is featured in specialty foods recognized as cultural heritage [44]. PTG grains have excellent storability and naturally store for 2 to 3 years without risk of infestation. PTG provides multifaceted significance spanning agriculture, economy, ecology, and culture in Pantiange, Yunnan. In recognition of its value, the Chinese Ministry of Agriculture and Rural Affairs designated PTG in 2022 as one of China’s Top Ten Prominent Agricultural Genetic Resources. However, its cultivation area shrank from 133 ha in the 1980s to less than 50 ha by 2020 [45].

Jumli Marshi from Nepal is one of the world highest-altitude rice cultivars cultivated above 3000 m [46]. Its paramount significance lies in its exceptional cold tolerance. It represents a vital genetic resource for international breeding programs aimed at developing climate-resilient rice varieties for temperature-variable regions worldwide. In contrast, PTG is unique in its specific adaptive combination; it has a tolerance to cold, waterlogged, and nutrient-poor peat soils of its native basin. Moreover, its distinctive black hull and red kernel phenotype are associated with high anthocyanin content. These characteristics are not commonly associated with high-altitude rice cultivations. Furthermore, PTG display nutritional richness in anthocyanins and specific amino acids, adding distinct quality advantages to its value. This highlights that PTG is both a unique genetic resource due to its specific adaptive traits and nutritional quality. Also, it is a broadly representative example of how landraces evolve locally optimized, complex trait packages, making them indispensable reservoirs of climate-resilient alleles. This distinction highlights the genetic and agronomic value of PTG that is representative of specialized landraces worldwide.

### 4.2. Conservation Strategies for PTG Landrace

**Figure 6:**
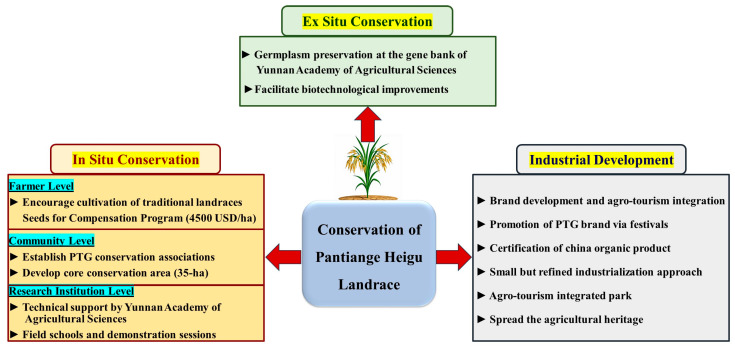
Integrated conservation and development strategies for Pantiange Heigu landrace.

Conservation of rice landraces is critical to preserve genetic diversity, cultural heritage, and agricultural resilience. Conservation strategies of PTG include strategies of in situ and ex situ conservation. In addition, conservation includes enhancing industrial development and brand value through targeted policies and market strategies (Figure 6). These approaches include collaborative efforts among farmers, research institutions, and local governments to maintain genetic purity, promote sustainable cultivation, and conserve germplasm for future breeding.

#### 4.2.1. In Situ Conservation

In situ conservation strategies rely on establishing collaborative networks among farmers, communities, and research institutions to effectively conserve PTG as follows:

1. At the farmer level: Implement a “Seeds for Compensation” program by encouraging the farmers economically with compensation payments to cultivate traditional landraces. The farmers receive support of 4500 USD (32,000 CNY) for every hectare cultivated by PTG. This model aligns with the Seed Systems model of FAO [47].
2. At the community level: Establish associations for PTG at the village level. Develop practical manuals, including standardized cultivation protocols and pest management. Additionally, designate a core conservation area of 35 hectares to restrict the cultivation of other rice varieties in this area. This is for the prevention of genetic contamination and preserves the seed purity of PTG.
3. At the research institution level: Yunnan Academy of Agricultural Sciences provides technical support for PTG cultivation. Conducting field schools and demonstration sessions to provide the farmers with advanced cultivation technologies. Furthermore, screening elite stress-tolerant and stable plants to maintain the genetic stability of the landrace.

#### 4.2.2. Ex Situ Conservation Strategy

Ex situ conservation is critical to preserve the genetic diversity of PTG. This approach involves the systematic collection and preservation of PTG germplasm in the gene bank of Yunnan Academy of Agricultural Sciences. Seeds conserved under controlled low-temperature and low-humidity conditions preserve PTG. This ensures long-term viability and availability for future breeding programs. The conserved seeds are an important resource for genetic characterization, molecular breeding, and biotechnological improvements of PTG. Therefore, the ex situ strategy contributes to conserving the uniqueness of PTG and enhances its role for future rice breeding activities. This collaboration between local communities and research organizations supports effective conservation of rice landraces. This integration supports resilience against environmental changes and agricultural challenges.

The conservation of upland rice landraces in the Cordillera Region of the Philippines presents a contrasting but complementary model to the PTG case [48]. It demonstrates that effective conservation strategies must be holistic and community-led. Moreover, it must involve integrating in situ cultivation with the protection of associated knowledge systems and cultural values that sustain these landraces. This case powerfully illustrates that for many traditional landraces, their resilience is rooted as much in cultural importance as in their genetic traits. Unlike the PTG model, which benefits from structured policy frameworks and direct research-institution support, the Cordillera case emphasizes how robust community governance can sustain agrobiodiversity. This indicates that effective conservation models can develop from diverse foundations ranging from formal, research-integrated systems to informal, culturally reinforced networks. Although both depend on aligning conservation goals with the encouragement and knowledge of local custodians. This demonstrates a vital dimension for designing comprehensive and sustainable conservation frameworks.

#### 4.2.3. Industrial Development and Value Enhancement

Over the past decade, Pantiange has built the PTG brand with strong support from local governments. The festival of “Three-River-Hinterland Weixi Pantiange Heigu Cultural Tourism” in 2016 established October 2nd as an annual event to support local cultural visibility. In 2019, the government attracted external operators such Xianggelila Yongchang Agricultural Science and Technology Service Co., Ltd. This establishment initiated PTG cultivation based on organic and EU standards. Despite facing challenges of COVID-19 pandemic, PTG was selected in 2022 as one of the Top Ten Prominent Agricultural Genetic Resources in China. In the future, the government will continue to support the branding and promotion of PTG. Efforts will focus on the inclusion of PTG in important catalogues such as “China Nationally Important Agricultural Heritage Systems” and “Globally Important Agricultural Heritage Systems.” Successful designation in these programs can access international funding, elevate the global recognition of PTG conservation, and facilitate the dissemination of its unique cultural heritage worldwide. Moreover, the efforts will focus on adding PTG to the National Geographical Indication Protection Product list. This will considerably enhance brand recognition, protect product authenticity, and improve market acceptance. Furthermore, this will exploit the pristine peat soils of Pantiange to have the certification of China Organic Product. In this context, Heilongjiang Wuchang Daohua previously achieved the best pricing following organic certification and could be a successful model for PTG. Adopting the industrialization strategy of “Small but Refined” can promote the conservation of PTG through its sustainable development.

Pantiange has historical importance as the highest-altitude producer of PTG landrace and has a unique agricultural heritage. This importance supports Pantiange in establishing innovative agro-tourism development. The government initiated the town project for the highest altitude rice cultivation by developing an agro-tourism integrated park (agriculture and tourism). This agro-tourism integrated park focuses on the PTG brand and establishes related industries such as livestock and poultry farming. The activities in this park include rice–fish and rice–duck farming systems to involve tourists in rice cultivation and harvest. Moreover, suggested pathways include developing cultural IP by animated short films about PTG via social media to attract younger audiences. Also, introducing creative products such as PTG boxes containing seeds, planting guides, and cultural cards may encourage the engagement of the consumers.

The strategy of developing a protected brand identity for PTG reflects a globally recognized model for conserving agrobiodiversity by enhancing its economic and cultural value. This approach is supported by international frameworks such as the FAO guide on Linking People, Places and Products [49]. It calls for using geographical indications and quality-linked branding to protect traditional agro-food products. These mechanisms formally link product unique qualities to its specific place of origin and cultural heritage. This creates a market premium that encourages the farmers to continue cultivating traditional landraces. Prominent examples include Thailand “Khao Dawk Mali 105” (Thai Hom Mali or Jasmine Rice) and the “Basmati” rice of India. Both have achieved formal geographical indication status and are legally linking their unique sensory qualities to their specific regions of cultivation and traditional knowledge. This protection has transformed them into globally recognized premium products, generating substantial market value and providing direct economic incentives for farmers to sustain cultivation. Consequently, the conservation of these genetic resources is driven by market demand and cultural pride. This demonstrates that strategic branding is a powerful tool for translating the genetic and cultural value of landraces into sustainable economic and conservation outcomes.

## 5. Lessons from PTG Landrace

The case study of PTG landrace provides valuable information that can enhance the strategies of conservation and sustainable development for other landraces facing similar climatic and socio-economic pressures.

➢ The case of PTG demonstrates the economic and cultural significance of landraces besides their breeding values. Enhancing local branding, developing value-added products, and linking landrace cultivation to heritage can create sustainable opportunities to continue cultivation and support agrobiodiversity conservation.
➢ Multi-sectoral collaboration between researchers, policymakers, local communities, and industry stakeholders is very important to achieve effective frameworks for conservation and sustainable development of rice landraces. Effective collaboration is essential to overcome challenges of funding, market access, and policy support.
➢ The combination of in situ conservation to maintain landraces in their native farming systems with ex situ conservation in seed banks is an important strategy for genetic resource management. Therefore, this dual approach is essential to balance conservation and availability for future breeding and research.

Consequently, the integrated conservation and development model demonstrated by the PTG landrace offers critical and transferable insights. The PTG case study reveals that successful scaling of community-based agrobiodiversity initiatives rests on applying universal principles, linking conservation directly to local benefits, empowering community-led governance, and leveraging the unique genetic and cultural value of the landrace. These principles provide a robust framework and a principle-based pathway adaptable to any region possessing a distinct local crop variety. The central lesson is that successful transfer is a process of guided adaptation, where external institutions, research bodies, and governments act as facilitators. Their role is to enable communities to prove the value of their resources, strengthen local institutions, and connect to supportive networks and markets.

## 6. Future Activities

Future directions for conservation and sustainable utilization of rice landraces should employ advanced technologies. Application of genomic tools such as whole-genome sequencing, genome-wide association studies, and marker-assisted selection is crucial to characterize the adaptive characteristics in rice landraces. Understanding the molecular basis of stress tolerance, nutritional quality, and yield stability could significantly improve rice breeding for climate resilience. In addition, enhancing ex situ conservation by comprehensive phenotyping, genotyping, and bioinformatics analysis could support global research collaborations. Moreover, developing community seed systems in in situ conservation can enhance the long-term preservation of landrace populations.

Economic encouragements by compensation payments, product branding, and organic certification can support conservation and sustainable development of rice landraces. Moreover, policy frameworks should list landrace conservation in the national agricultural development plans. Establishing collaborative network among policymakers, farmers, and researchers is essential to support conservation and sustainable utilization of rice landraces. The impacts of climate change on landrace performance and their adaptive strategies should be studied. This will ensure that the conservation efforts remain forward-looking. These activities will effectively support conservation and sustainable utilization of rice landraces to face the challenges of climate change worldwide.

## 7. Conclusions

Rice landraces are essential genetic resources to improve the resilience of global rice production systems. As demonstrated in this review, their wide genetic diversity reflects centuries of adaptation to diverse agro-ecological conditions. The evidence confirms that this diversity translates into significant contributions for improving tolerance to biotic and abiotic stresses and for enhancing nutritional quality. Consequently, conservation and sustainable utilization of these genotypes are essential to face environmental challenges under current climate change. The conservation and sustainable use of rice landraces must be recognized and supported as a vital resource that reinforces long-term food system resilience. Policy frameworks at national and international levels must explicitly value and fund integrated conservation strategies. This funding could be provided through dedicated mechanisms such as support for on-farm cultivation, and sustained investment in public seed bank systems, and also, the inclusion of agrobiodiversity indicators in national agricultural development plans. Such policy commitment is the essential enabling condition that will allow the technological advances and community-based models to be scaled and sustained. The Pantiange Heigu is a unique high-altitude rice variety adapted to severe climatic and soil conditions. PTG has significant genetic, nutritional, and cultural value. Its conservation model demonstrates the critical importance of integrated approaches that combine in situ cultivation with ex situ gene bank preservation, supported by targeted policy and economic incentives. Therefore, as this review has explored, establishing a collaborative network among farmers, communities, policymakers, and research institutions is essential to conserve landraces globally. Furthermore, exploiting advanced genomics tools with traditional knowledge can provide an effective strategy for conserving these genetic resources. Through continued research, conservation, and sustainable utilization of landraces, rice breeding can cope with climate change to ensure food security for future generations.

## Data Availability

No new data were created or analyzed in this study.

## Abbreviations

The following abbreviations are used in this manuscript:

PTG	Pantiange Heigu Landreace
QTL	Quantitative Trait Loci
GWAS	Genome-Wide Association Studies
SNP	Single-Nucleotide Polymorphism
SSR	Simple Sequence Repeat
R genes	Resistance genes
FAO	Food and Agriculture Organization of the UN
USD	United States Dollar
EU	European Union
COVID-19	Coronavirus Disease 2019
